# Clinical application of modified Crain classification in the Design of Anterior Cruciate Ligament Reconstruction with remnant preservation

**DOI:** 10.1186/s12891-022-05912-7

**Published:** 2022-12-05

**Authors:** Zheng Wang, Hai-bing Tao, Yu Wang, Bin Liu, Wen-feng Han, Liang-bi Xiang

**Affiliations:** Department of orthopedics, General Hospital of Northern Theater Command, Shenyang, 110016 China

**Keywords:** Modified Crain classification, Anterior cruciate ligament, Reconstruction with remnant preservation

## Abstract

**Background:**

To investigate the clinical application of modified Crain classification in anterior cruciate ligament (ACL) reconstruction (ACLR) with remnant preservation.

**Methods:**

The subjects were 70 patients with ACL injury who underwent ACLR from May 2016 to June 2018, and their general data were recorded. They were randomly divided into modified remnant-preserved ACLR group (group M, *n* = 35) and non remnant-preserved ACLR group (group N, n = 35). ACLR program with remnant preservation was designed based on modified Crain classification in group M, while ACL remnants were completely cleaned during ACLR in group N. Subsequently, the two groups were compared in terms of operation time, complications, as well as Lysholm score, international knee documentation committee (IKDC) score and positive rate of Lachman test of knee joint before operation and at 3, 6 and 12 months after operation.

**Results:**

Both the groups showed good postoperative efficacy, and none had complications like limited knee extension or cyclops lesion. The comparison results found that group M (72.49 ± 7.64 min) required longer operation time than group N (66.06 ± 6.37 min) (*P* < 0.05). Lysholm score and IKDC score at 3, 6 and 12 months after operation in the two groups were significantly higher than those before operation (*P* < 0.05); group M had higher Lysholm score and IKDC score at 3 months and 6 months after operation compared with group N (P < 0.05). Additionally, the positive rate of Lachman test at 3, 6 and 12 months after operation in both groups was significantly lower than that before operation (*P* < 0.05), but there was no significant difference between group M and group N.

**Conclusion:**

With the modified Crain classification, many remnant-preserved reconstruction techniques can be rationally used to completely preserve the remnant ligament tissue during operation and improve knee joint function and joint stability with few complications.

## Background

Anterior cruciate ligament (ACL) injury is the most common injury of the knee joint [1]. Once torn, the ACL cannot repair itself due to lack of blood supply or poor intra-articular environment, resulting in meniscus injury and even joint degeneration. Most believe that early rehabilitation after ACL reconstruction (ACLR) can optimize the therapeutic outcomes in terms of recovering knee joint function and maintaining its mechanical stability [2]. ACLR has become a conventional treatment for ACL injury, but the intraoperative management of remnant ligament tissue remains controversial [3–5]. With the in-depth study, some scholars support reconstruction with remnant preservation [6–11], for several reasons. First, the preserved remnant ligament tissue can accelerate the biological healing of the graft by enhancing cellular proliferation and revascularization, contributing to satisfactory clinical results [6, 10, 11]. Secondly, proprioceptors are enriched in the synovium covering the surface of remnant ligament tissue and facilitate the rapid recovery of neuromuscular control and motor function of the knee joint [9, 12–14]. Thirdly, the preservation of remnant ligament tissue helps to maintain the stability of the knee joint and thus reduces the early mechanical load of grafts [15, 16]. However, traditional remnant-preserved reconstruction is difficult to operate and lacks meticulous evaluation of the stump, and worse, it can lead to complications such as limited knee extension and cyclops lesion [17–19]. Many new techniques have emerged to surgical effect of remnant-preserved reconstruction in recent years, but there is a lack of appropriate guidance on the clinical application of ACLR with remnant preservation.

Classifying the morphology of ACL remnants is the basis for the application of remnant-preserved reconstruction [20]. Crain et al. proposed a classification [21] of remnant ACL according to the patterns of its stump [21], and further the modified Crain classification was developed [22]. We applied the modified Crain classification in designing ACL remnant-preserved reconstruction program to improve the surgical effect. This paper attempts to explore the clinical application of the modified Crain classification in the design of remnant-preserved ACLR operation program, providing a new option and reference for optimization of the operation.

## Materials and methods

### Patient collection

We applied the modified Crain classification in designing remnant-preserved ACLR program to improve the surgical effect. The subjects were patients with ACL injury who underwent ACLR from May 2016 to June 2018. This study conformed to the requirements of the ethics committee of General Hospital of Northern Theater Command (Y^− 2021^-027), and all patients signed an informed consent.

Inclusion criteria: 1) ACL injury met the standard of reconstruction; 2) Remnant ligament tissue with no obvious atrophy under arthroscopy; 3) No previous knee surgery. Exclusion criteria: 1) Complicated by multiple ligament injuries and fractures; 2) Complicated by obvious lesions of the knee joint before ACL injury; 3) ACL injury in both knees.

Patients were randomly divided into group M (*n* = 35) and group N (n = 35). Patients in group M received remnant-preserved ACLR based on modified Crain classification, while those in group N were given ACLR with complete clearance of the remnants.

### Modified Crain classification

**Fig. 1:**
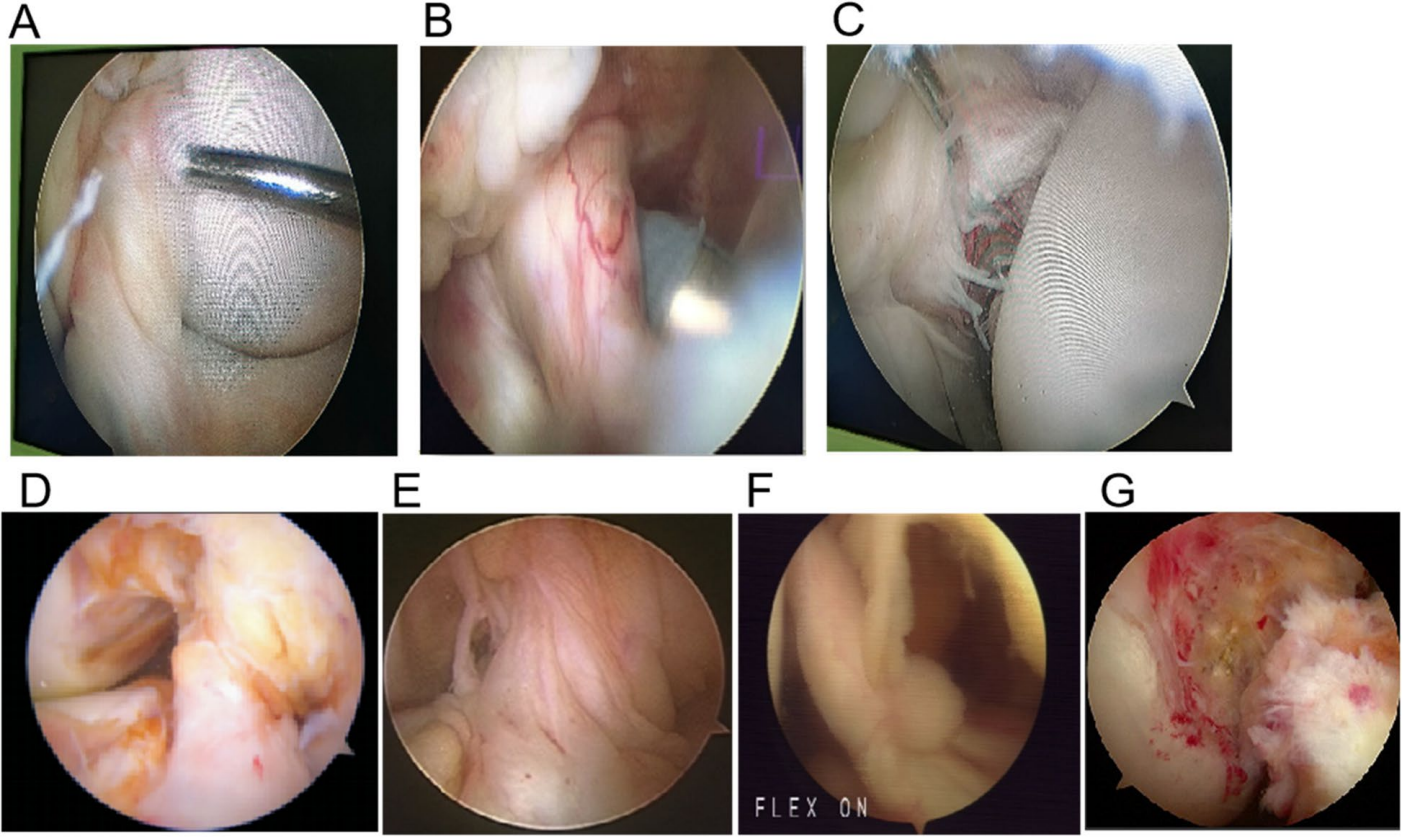
Modified Crain classification of remnant ligament tissue. **A**: Groups Ia anterior cruciate ligament (ACL) remnant; **B**: Group Ib ACL remnant; **C**: Group Ic ACL remnant; **D**: Group IIa ACL remnant; **E**: Group IIb ACL remnant; **F**: Group IIc ACL remnant; **G**: Group IId ACL remnant

Groups I ACL remnant referred to partial rupture of the ACL, including groups Ia, Ib, and Ic (Fig. 1A-C). In group Ia, the anteromedial (AM) bundle was well preserved but the posterolatera (PL) bundle ruptured. In group Ib, the PL bundle was well preserved and the AM bundle had a rupture. In group Ic, both PL bundle and AM bundle ruptured, but ligamentous continuous fibers were still preserved in the normal attachment of ACL to the femur.

Groups II ACL remnant referred to complete rupture of the ACL with no ligamentous continuous fibers in the normal attachment of ACL to the femur, including groups IIa, IIb, IIc, and IId (Fig. 1D-G). In group IIa, ACL remnant bridged the posterior cruciate ligament and tibia, without normal attachment of the ACL to the intercondylar notch. In group IIb, ACL remnant bridged the roof of the intercondylar notch and tibia, without normal attachment of the ACL to the femur. In group IIc, ACL remnant bridged lateral wall of the intercondylar notch and tibia, and healed to the medial wall of lateral femoral condyle. In group IId, no ACL remnant bridged the tibia and either the femur or the posterior cruciate ligament.

### Operation program

For group M, after successful anesthesia, patients were placed in the supine position with the knee flexed at 90°. The bilateral patellar tendon approach was made for the insertion of an arthroscope and subsequent inspection of the morphology of ACL remnants. The remnants were classified using the modified Crain classification; then single-bundle reconstruction with remnant preservation was used for groups Ia and Ib while double-bundle reconstruction with remnant preservation for groups Ic, IIa, IIb, IIc. Autologous semitendinosus–gracilis tendon was selected as the graft. Using the guide pin and a drill, one tunnel through the femur and one tunnel through the tibia were created. An Endobutton anchored the upper end of the graft at the femoral site and an interference screw stabilized the lower end at the tibial side. Tendons ouside the tibial tunnel were fixed with spiked ligament staple. Afterwards, suturing was performed, with groups Ia, Ib, Ic, IIc, IId ACL remnants sutured using long guidewire [23, 24], and groups IIa and IIb treated with moderate radiofrequency release followed by suture with a suture hook [25].

For group N, ACL remnants at the femoral and tibial sides were completely cleaned during the surgery. The central point of the femoral anatomical footprint was used as an anchor point at the femoral side, while the slightly anterior position of the tibial footprint center as an anchor point at the tibial side. The bony landmarks were fully exposed to determine the accurate femoral and tibial anchor points. The rest procedures were the same as those in the group M.

After operation, functional exercise of quadriceps femoris and ankle pumps were performed. The patient’s knee was fixed and extended with the brace for 10 days, and the flexion angle was increased by 30° every week until reaching 120°. Post-operative reexaminations by the surgeon, included magnetic resonance imaging (MRI, Philips 1.5 Tesla Intera), three-dimensional computed tomography (CT, General Electrics Lightspeed VCT 16, USA), and radiography of the knee. Three-dimensional CT images of the femoral tunnel were presented in the longitudinal and transverse planes.

### Postoperative follow-up

The patients were followed up at 3 months, 6 months and 12 months after operation. Lysholm score and international knee documentation committee (IKDC) score were recorded to evaluate the recovery of knee joint function, and Lachman test was performed to assess the joint stability. Additionally, the incidence of complications was recorded and compared between the two groups.

The Lysholm score [26] and IKDC score [27] are two 100-point scoring systems for evaluating knee function, and higher score indicates better knee function. A positive result of Lachman test of the knee joint is evidence of an unstable knee joint and an excessive forward movement of the tibia compared to the normal; a negative result indicates that the knee joint is stable [28].

### Statistical analysis

SPSS 22.0 software was used to process the data. Measurement data were expressed as mean ± standard deviation (SD), with independent-samples t-test for comparison between the two groups. Enumeration data were expressed as n (%), with χ^2^ test for comparison. *P* < 0.05 indicated the difference was significant.

## Results

### General data of patients

There were 35 patients in group M, including 28 males and 7 females, aged 20 to 43 years (average age: 29.31 ± 5.42 years). And there were 35 patients in group N, including 26 males and 9 females, aged 20 to 42 years (average age: 28.89 ± 5.76 years). No significant difference was identified in sex and age between the two groups. However, the operation time of group M (72.49 ± 7.64 min) was significantly longer than that of group N (66.06 ± 6.37 min) (*P* < 0.001).

### Comparison of efficacy between the two groups

**Fig. 2:**
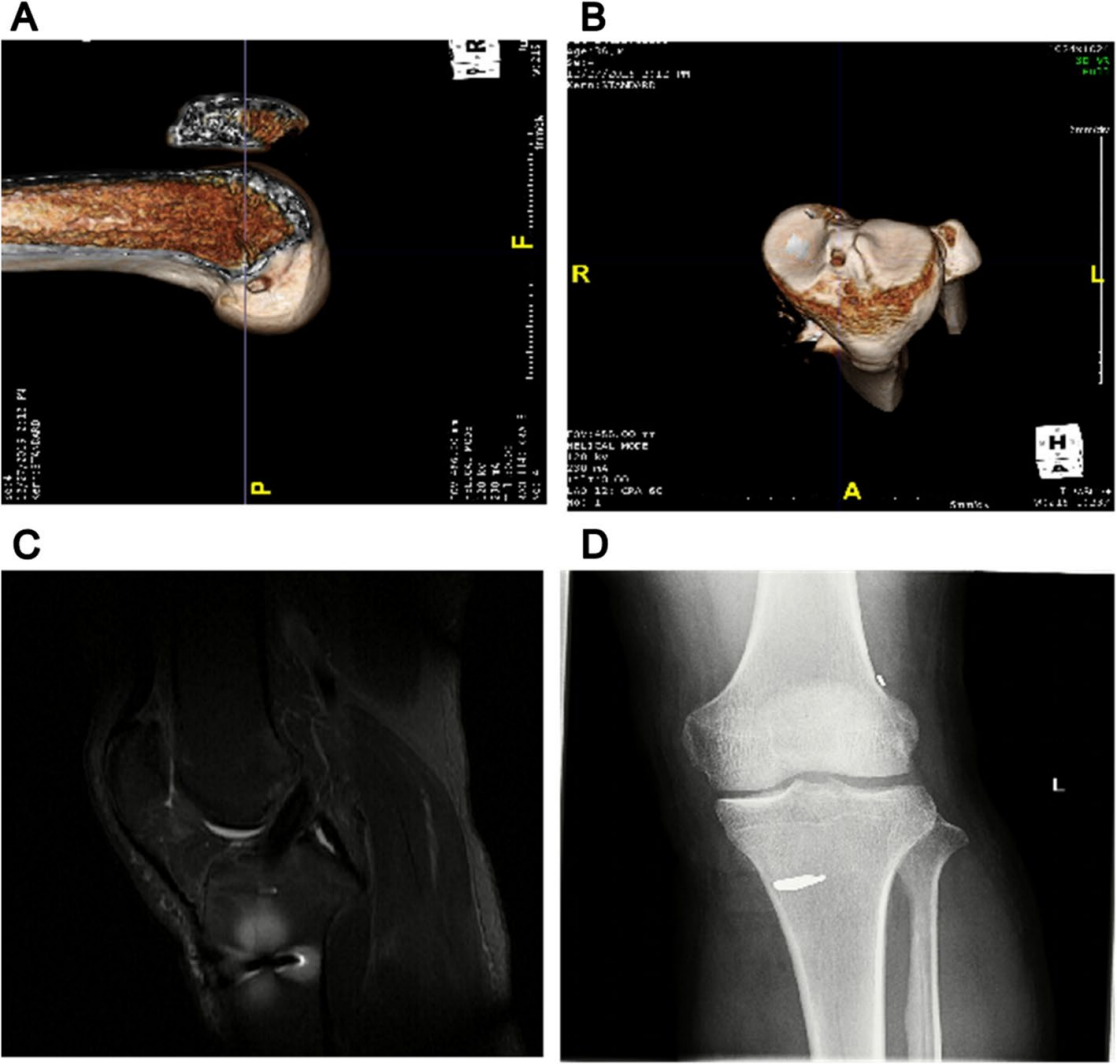
Postoperative imaging examination of patients. **A-B**: Postoperative three-dimensional CT images of femoral tunnel of group M patients in longitudinal (**A**) and transverse (**B**) planes; **C**: Postoperative magnetic resonance imaging of ligament signal of group M patients; **D**: Postoperative X-ray scanning of screw fixation of group M patients

Further post-operative three-dimensional CT, MRI, and X-ray scans of the patients in the group M showed no significant enlargement of the bone tunnels (Fig. 2A-B). Their ligament signals were intact and continuous, with no manifestations of cyclops lesion (Fig. 2C). Additionally, there were no complications such as screw loosening (Fig. 2D).

All patients were followed up at 3, 6 and 12 months after operation and post-operative efficacy was compared between the two groups. As revealed by the intragroup comparison results (Table 1 and Table 2), Lysholm score and IKDC score in the two groups during follow-up were significantly higher than those before operation (*P* < 0.05). At 3 and 6 months after operation, the intergroup comparison showed that group M had higher scores (*P* < 0.01), but the two groups had no significant difference at 12 months after operation (*P* > 0.05). The positive rate of Lachman test in both groups at these three postoperative time points was significantly lower than that before operation (*P* < 0.01), but no significant difference was found between the two groups in the positive rate of Lachman test before operation and at postoperative follow-up (*P* > 0.05; Table 3).

**Table 1:** Comparison of Lysholm function score of knee joint between two groups (points)

Group	Case (n)	Pre-operation	3 months after surgery	6 months after surgery	12 months after surgery
Group M	35	48.83 ± 4.11	81.31 ± 3.60*	86.57 ± 3.05*	92.69 ± 2.52*
Group N	35	49.26 ± 3.54	75.34 ± 4.10*#	82.17 ± 3.21*#	91.91 ± 2.59*
t value	–	−0.468	6.477	5.875	1.263
^#^*P* value	–	0.640	0.000	0.000	0.211

Data was expressed as mean ± SD. *P<0.05 vs. Pre-operation, intragroup comparison; #*P*<0.05 vs. Group M, intergroup comparison. Group M, patients received remnant-preserved anterior cruciate ligament reconstruction (ACLR) based on modified Crain classification; group N, patients given ACLR with complete clearance of the remnants

**Table 2:** Comparison of IKDC score of knee joint between two groups (points)

Group	Case (n)	Pre-operation	3 months after surgery	6 months after surgery	12 months after surgery
Group M	35	48.29 ± 3.89	80.51 ± 3.62*	87.29 ± 2.71*	93.06 ± 2.52*
Group N	35	49.54 ± 2.87	73.66 ± 3.57*#	81.94 ± 3.40*#	92.37 ± 2.55*
t value	–	−1.537	7.980	7.280	1.133
^#^*P* value	–	0.254	0.000	0.000	0.261

Data was expressed as mean ± SD. * P<0.05 vs. Pre-operation, intragroup comparison; #*P*<0.05 vs. Group M, intergroup comparison. Group M, patients received remnant-preserved anterior cruciate ligament reconstruction (ACLR) based on modified Crain classification; group N, patients given ACLR with complete clearance of the remnants. IKDC, international knee documentation committee

**Table 3:** Positive rate of Lachman test in the two groups

Group	Case (n)	Pre-operation	3 months after surgery	6 months after surgery	12 months after surgery
Group M	35	35 (100.0)	2 (5.7)^*^	2 (5.7)^*^	3 (8.6)^*^
Group N	35	35 (100.0)	3 (8.6)^*^	3 (8.6)^*^	5 (14.3)^*^
χ^2^	–	–	0.000	0.000	0.141
*P*^*a*^	–	–	1.000	1.000	0.707

Data was expressed as n (%). *P<0.05 vs. Pre-opration. Group M, patients received remnant-preserved anterior cruciate ligament reconstruction (ACLR) based on modified Crain classification; group N, patients given ACLR with complete clearance of the remnants

## Discussion

Patients with ACL injuries were included in this study, and two treatment modalities, remnant-preserved ACLR based on modified Crain classification (group M) and ACLR with complete removal of remnants (group N), were applied to the two groups of patients, respectively. We found that after the former treatment, patients had significantly improved Lysholm score and IKDC score, increased positive rate of Lachman test, and few complications.

Reconstruction with remnant preservation has been the research focus in ACLR. Although many scholars believe remnant-preserved ACLR has numerous potential advantages, remnant preservation shows no superiority in the recovery of knee joint function and joint stability, and will bring some problems [18, 19]. For example, preserved remnant ligament tissue is mostly structurally irregular and tends to present a floating state after grafting, which often increases the post-operative incidence of cyclops lesion and may affect the extension of the knee joint [11, 29]. In addition, preserved remnants will affect the view under arthroscopy during ACLR, thus increasing the difficulty of surgery, prolonging the operation time, and possibly leading to wrong tunnel positioning [30]. With the advance of new techniques of remnant-preserved reconstruction, these problems are solved to some extent and more scholars consider to completely preserve remnants, but such reconstruction is still lack of systematic design in clinical application.

Some studies have shown that classifying the morphology of ligament remnants can guide the reasonable application of remnant-preserved reconstruction technique. In this study, for groups Ia and Ib ACL remnants, the remaining bundle can be completely preserved by single-bundle reconstruction with the remnant-preserving technique. The undamaged vascular and neural structures in the preserved bundle can accelerate the biological healing of the graft and the recovery of neuromuscular control [31–33]. Double-bundle reconstruction with the remnant-preserving technique can completely preserve the groups Ic, IIa, IIb, IIc ACL remnants, facilitating the healing of the graft and early reinnervation of nerve fibers [34]. Additionally, we used the corresponding suture technique for different ACL remnants in group M, which could better restore the integrity of remnant ligament tissue during the operation and caused no significant increase in operation time [22]. Our results demonstrated that although group M had a longer operation time, but the increase of average operation time was only 6.4 min.

Patients undergoing ACLR have better IKDC subjective scores in a previous study [35]. Similarly, we found that both ACLR programs could significantly improve the knee function of patients, with better outcomes in group M at 3 and 6 months after operation. Both reconstruction methods could also markedly improve knee joint stability. However, in most existing studies, remnant preservation is not associated with better joint function in the early postoperative period. The differences can be explained by the modified Crain classification used in our study that improves the outcome of ACLR. Moreover patients developed no complications such as limited knee extension and cyclops lesion, and their bone tunnels were also in a good state after remnant-preserved ACLR based on modified Crain classification. Previously, the preservation of remnant ligament tissue has negative effects on ACLR difficulty, operation time, and incidence of complications [16, 17]; such results may be due to the fact that the traditional ACLR was not systematically designed and the specific situation of remnant ACL was not considered. As a result, what is often preserved is the remnants with incomplete structure, and such remnants have no contribution to the improvement of knee joint function [31] but possibly bringing complications. In this study, the remnant-preserved ACLR program was designed based on modified Crain classification. This program could reasonably apply remnant-preserved reconstruction techniques to restore the integrity of ACL remnant, thereby facilitating rapid postoperative healing and early regeneration of neurovasculature in patients and contributing to the early recovery of knee joint function [4, 5, 31].

There are some limitations of this study. First, the sample size was small, with only 35 cases in each group. This number of samples was sufficient for group N with the same surgical access, but insufficient for group M having Crain classification (groups Ia-IId). Secondly, patients in group M underwent different surgical approaches according to the classification, but the results of different approaches were not compared. In addition, more experimental indicators were not measured, such as proprioceptive testing. This all needs the inclusion of more patients for further analysis.

## Conclusion

With the modified Crain classification, many remnant-preserved reconstruction techniques can be rationally used, achieving complete preservation of the remnant ligament tissue during operation and improvement of knee joint function and joint stability, with few complications.

## Data Availability

The data that support the findings of this study are available from the corresponding author upon reasonable request.

## Abbreviations

ACL: Anterior cruciate ligament
ACLR: Anterior cruciate ligament reconstruction
CT: Computed tomography
AM: Anteromedial
PL: Posterolatera
MRI: Magnetic resonance imaging
IKDC: International knee documentation committee
